# Floristic homogenization of South Pacific islands commenced with human arrival

**DOI:** 10.1038/s41559-023-02306-3

**Published:** 2024-01-15

**Authors:** Nichola A. Strandberg, Manuel J. Steinbauer, Anna Walentowitz, William D. Gosling, Patricia L. Fall, Matiu Prebble, Janelle Stevenson, Janet M. Wilmshurst, David A. Sear, Peter G. Langdon, Mary E. Edwards, Sandra Nogué

**Affiliations:** 1 School of Geography and Environmental Science, University of Southampton, Highfield, Southampton, UK; 2 Bayreuth Center of Ecology and Environmental Research (BayCEER) and Bayreuth Center for Sport Science (BaySpo), University of Bayreuth, Bayreuth, Germany; 3 Department of Biological Sciences and Bjerknes Bergen, Bergen, Norway; 4 Department of Biogeography, University of Bayreuth, Bayreuth, Germany; 5 Department of Ecosystem and Landscape Dynamics, Institute for Biodiversity and Ecosystem Dynamics, University of Amsterdam, Amsterdam, The Netherlands; 6 Department of Geography and Earth Sciences, University of North Carolina at Charlotte, Charlotte, NC USA; 7 School of Earth and Environment, University of Canterbury, Christchurch, New Zealand; 8 School of Culture, History and Language, ANU College of Asia and the Pacific, Australian National University, Canberra, Australian Capital Territory Australia; 9 ARC Centre of Excellence for Australian Biodiversity and Heritage, Australian National University, Canberra, Australian Capital Territory Australia; 10 Long-term Ecology Laboratory, Manaaki Whenua-Landcare Research, Lincoln, New Zealand; 11 Universitat Autònoma de Barcelona, Bellaterra (Cerdanyola del Vallès), Catalonia, Spain; 12 CREAF, Bellaterra (Cerdanyola del Vallès), Catalonia, Spain

**Keywords:** Macroecology, Palaeoecology, Tropical ecology, Biogeography

## Abstract

The increasing similarity of plant species composition among distinct areas is leading to the homogenization of ecosystems globally. Human actions such as ecosystem modification, the introduction of non-native plant species and the extinction or extirpation of endemic and native plant species are considered the main drivers of this trend. However, little is known about when floristic homogenization began or about pre-human patterns of floristic similarity. Here we investigate vegetation trends during the past 5,000 years across the tropical, sub-tropical and warm temperate South Pacific using fossil pollen records from 15 sites on 13 islands within the biogeographical realm of Oceania. The site comparisons show that floristic homogenization has increased over the past 5,000 years. Pairwise Bray–Curtis similarity results also show that when two islands were settled by people in a given time interval, their floristic similarity is greater than when one or neither of the islands were settled. Importantly, higher elevation sites, which are less likely to have experienced human impacts, tended to show less floristic homogenization. While biotic homogenization is often referred to as a contemporary issue, we have identified a much earlier trend, likely driven by human colonization of the islands and subsequent impacts.

## Abstract (web summary)

Exploring pollen records from 13 South Pacific islands, the authors find that floristic homogenization commenced following human arrival beginning around 3,000 years ago.

## Main

Biotic homogenization threatens global biodiversity, as the increasing similarity of species composition among regions leads to an overall decline of inter-regional diversity^1,2^. Homogenization is often linked to anthropogenic impacts such as habitat loss and range expansions of generalist introduced taxa, which can lead to range contractions and/or population declines of native/endemic species^3–6^. Increased homogeneity may also affect ecosystem function and services and thus (socio)ecosystem resilience in the face of further anthropogenic impacts^7,8^.

In many ways, islands are ideal models for studying biodiversity changes^9^. Insular biotas are particularly vulnerable to human-mediated drivers that are known to be causes of the biotic homogenization process, such as species introductions, habitat loss and climate change^10–14^. In addition, island ecosystems have higher proportions of endemics than mainland areas^15,16^, thus making them important habitats and high priorities for conservation^10^. In the biogeographical realm of Remote Oceania, many plant species have threatened status, although the scale of the problem is still unclear, as only ∼3% of species have been assessed by the International Union for Conservation of Nature^17^. Due to the threatened status of many plant species, it is likely that Pacific islands have been susceptible to floristic homogenization in both the past and present (for example, refs. ^18–20^).

Many studies of biotic similarity rely on published lists of native, non-native, extinct or extirpated taxa to delineate a baseline for biodiversity composition. Such lists limit the time frame for setting a baseline to a few centuries, and they exclude unrecorded introductions or extirpations (for example, refs. ^20–23^). Fossil datasets have the potential to extend these timescales and capture previously unrecorded introductions or extirpations^24–32^.

Remote Oceania was colonized by people in two broad migrations ∼3,300–2,700 and ∼1,000–700 years ago (see Supplementary Table 1 and references therein). Studies of the islands have provided an abundance of information, both palaeoecological and archaeological, allowing us to observe and quantify trends in floristic similarity on a longer temporal scale and to observe human settlement and activity. By analysing 15 fossil pollen records from Remote Oceania encompassing the past 5,000 years and a west-to-east gradient of 8,300 km, we quantify the taxonomic similarity of island floras over time. We use two methods for standardizing taxon names: standardization-1, which preserves the lowest level of identification, and standardization-2, which uses a higher level of taxonomic identification. We use Bray–Curtis similarity for the pairwise similarity analyses^33^.

## Results

**Fig. 1:**
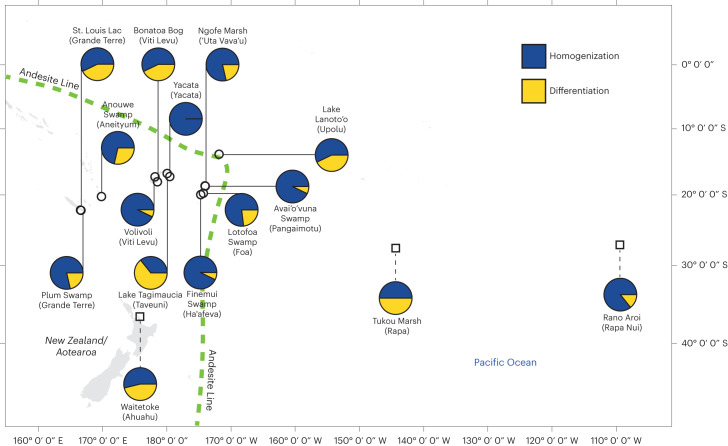
Homogenizing and differentiating trends in floristic similarity for each site. Proportions for each site of Bray–Curtis similarity homogenizing trends (<0 slope coefficients) in blue and differentiating trends (>0 slope coefficients) in yellow over time based on the standardization-1 dataset. The Andesite Line is shown as a green dashed line. Circles with solid leader lines indicate sites settled ∼3,000 cal years BP, and squares with dashed leader lines indicate sites settled ∼700 cal years BP. Island names follow site names in parentheses. The *x* axis and *y* axis represent longitude and latitude, respectively.

We focus on the results of the standardization-1 approach, as it utilizes the lowest taxonomic level of identification. Out of a possible 105 slope coefficients (whole site-to-site comparisons over time), a majority indicate trends of homogenization for 13 of the 15 sites (Fig. 1), while Lake Tagimaucia (Taveuni), Waitetoke (Ahuahu), Tukou Marsh (Rapa), St. Louis Lac (Grande Terre), Bonatoa Bog (Viti Levu) and Lake Lanoto’o (Upolu) show the most differentiation, Yacata (Yacata Island), Avai’o’vuna Swamp (Pangaimotu) and Finemui Swamp (Ha’afeva) show the most floristic homogenization trends over time. Supplementary Fig. 11 shows the results of the analysis using the standardization-2 approach.

**Fig. 2:**
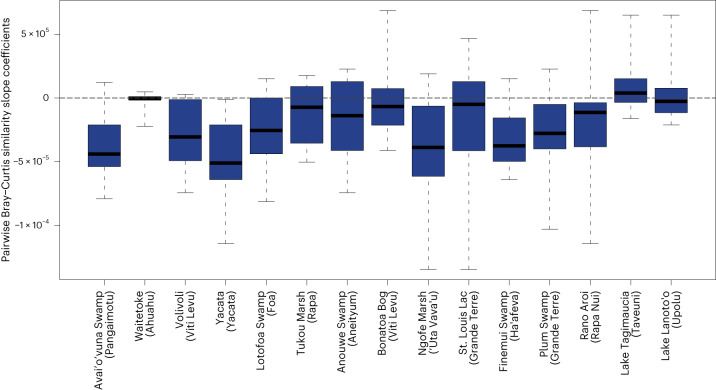
Floristic similarity trends for each site ordered by elevation. The direction and steepness of the floristic similarity trends is based on the standardization-1 dataset between sites (*n* = 14 site comparisons as we exclude comparisons within a given site) based on pairwise Bray–Curtis similarity slope coefficients. Sites are organized by elevation with the lowest (sea level) on the left to the highest on the right (760 m a.s.l.). Data points above the grey horizontal dashed line are differentiating trends and below this line are homogenizing trends. The black horizonal lines indicate the medians of the data for each site, and the blue bars encompass the first and third quantiles of the data. Whiskers extend to the maximum and minimum of the data.

The 105 whole site-to-site comparisons of the standardization-1 dataset over time offer another perspective: all sites except Lake Tagimaucia (Taveuni) have negative median slope coefficients, showing that they have become more similar to other sites (Fig. 2). Yacata (Yacata Island) has the lowest median slope coefficient and has therefore become the most similar to other sites over time. Avai’o’vuna Swamp (Pangaimotu), Ngofe Marsh (‘Uta Vava’u) and Finemui Swamp (Ha’afeva) also show strong floristic homogenization, while Waitetoke (Ahuahu) and Lake Lanoto’o (Upolu) show relatively low floristic homogenization. Supplementary Fig. 12 shows the results of the analysis using the standardization-2 approach.

**Fig. 3:**
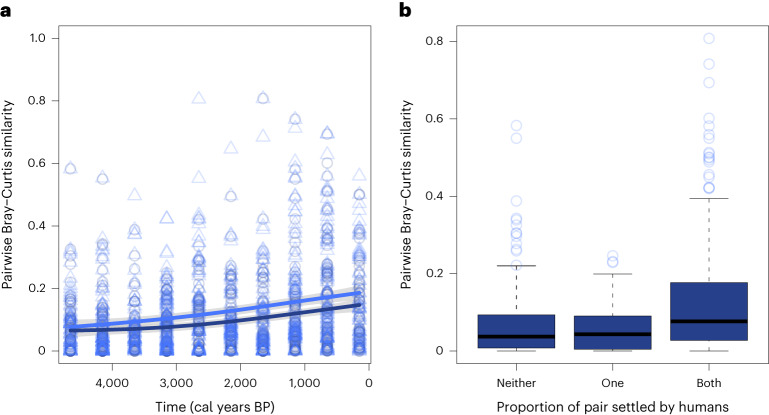
Floristic similarity trends over time and grouped by human occupancy for all sites. **a**, Non-parametric regressions were fitted using smoothing splines deploying package npreg^92^ with function ss (fit a smoothing spline). Smoothing splines showing pairwise Bray–Curtis similarity scores among all 15 sites on 13 islands, over the past 5,000 cal years BP. 1, greatest similarity; 0, lowest similarity. The dark blue line represents the standardization-1 dataset, and the lighter blue line represents the standardization-2 dataset. The grey shaded areas represent 95% confidence intervals. Open circles represent standardization-1 datapoints, and open triangles represent standardization-2 datapoints. **b**, Pairwise Bray–Curtis comparisons for the standardization-1 dataset grouped according to whether neither (*n* = 223), one (*n* = 430) or both (*n* = 157) islands were settled during a time interval. *n*, number of individual pairwise comparisons. The black horizonal lines represent the medians, and the box areas represent the first and third quantiles. The whiskers of the boxplot extend to the last data points within 1.5 times the range from first quantile to third quantile (interquartile range of the box).

The standardization-1 and standardization-2 smoothing splines show an increase in floristic similarity during the past 5,000 years (Fig. 3a). The smoothing splines show similarity values of 0.07 and 0.08 for the earliest time interval (4,900–4,400 calibrated years before present (cal years BP)) for standardization-1 and standardization-2, respectively. These values rise to 0.15 and 0.19 for the most recent time interval (400 cal years BP to the present), based on standardization-1 and standardization-2, respectively. The medians for comparisons in which people were not present on either island (0.04) or present on one island were similar (0.04) (Fig. 3b). However, when both sites were colonized by people, the median similarity was greater (0.08), indicating greater floristic homogeneity (Fig. 3b). See Supplementary Fig. 13 for results of standardization-2.

## Discussion

Given the large geographical area explored in this study (8,300 km west to east), it is striking to find a 5,000-year-long trend of floristic homogenization across 15 sites on 13 islands (Fig. 3a). In addition, when incorporating the potential human dimension to our analysis, the floristic homogenization process was enhanced when two islands were settled in the same time frame (Fig. 3b). As a whole, this information provides a strong indication that anthropogenic activities resulted in increased floristic similarity among the islands analysed.

### Non-anthropogenic drivers of floristic homogenization

Pairwise comparisons in which neither site or one site or island was settled show that similarity was relatively low (Fig. 3b). Dynamics in pre-human settlement composition are likely related to natural drivers such as sea level change, hydroseral development of wetlands and lacustrine vegetation, volcanism and other disturbances (for example, cyclones and droughts)^34–37^. These drivers may have an impact on biotic similarity in numerous biotas and habitats as they can affect a given species and thus alter the species pool in similar ways across different communities (for example, ref. ^38^). Finally, continued dispersal of pan-oceanic plant taxa should not be ignored as a contributing factor to floristic similarity on the islands. Indeed, on both western and eastern Pacific islands, bird dispersal of plant propagules is an important mechanism for both inter-island and intra-island dispersal (see ref. ^39^ for discussion on plant dispersal mechanisms). In this study, however, despite the range of natural drivers that could have affected the islands ecosystems, the results suggest that non-anthropogenic factors did not contribute to floristic homogenization, at least in a detectable way.

### Floristic homogenization linked to novel anthropogenic drivers

The trend towards floristic homogenization on settled islands (Fig. 3b) is likely caused by novel anthropogenic drivers. Within the novel drivers of change, burning (for example, as a part of agricultural activities), introduction of non-native taxa and extinctions or extirpations (of both fauna and flora) have been shown to impact island ecosystems^11,30,40^. First, the increased occurrence and intensity of disturbances is linked to anthropogenic modification of the islands’ landscapes, for example, as a consequence of slash and burn techniques^11,36,41^. It has also been shown that intense human impacts, such as urbanization and intensively managed agroforestry plantations, can lead to an increase in globally distributed taxa and therefore biotic homogenization^42,43^. Second, it is important to highlight that fossil pollen records from islands have also shown that non-native plant introductions increased within the past 1,000 years, suggesting potentially long-term presence of widely distributed species^30^. For example, non-native species introduced to multiple islands by people may have contributed to increased similarity among island ecosystems^30,44^. Last, there has been an anthropogenically driven extinction or extirpation of native or endemic plant families and genera (for example, *Pritchardia*) from Pacific islands^44–46^. The arrival of humans to Pacific islands is known to have coincided with many bird extinctions^47–51^. For example, it has been suggested that in Tonga, the decline of native birds and bats may have reduced the dispersal capabilities of many trees with large seeds, such as *Calophyllum inophyllum*, *Cerbera odollam*, *Planchonella garberi*, *Planchonella* *membranacea*, *Pometia pinnata*, *Syzygium quadrangulatum, Syzygium* *richii* and *Terminalia catappa*^48,52–54^. Predation of seeds by rodents introduced by both Polynesians and Europeans is also a suggested cause for the decline of many plant taxa in Tonga and Rapa Nui (Easter Island)^48,55^. Therefore, it cannot be ruled out that the increase in similarity between islands has also been driven by a decrease in trees with large, animal-dispersed fruits^56^.

As Bray–Curtis similarity is sensitive to changes in abundant taxa^57^, it is important to consider what these taxa are. Before colonization of any of the islands (3,000 and 700 cal years BP), Euphorbiaceae was the most abundant taxon (comprising 7% mean value of the pollen sum). After colonization, Cyperaceae and Poaceae became the most abundant taxa (17% and 10% mean values, respectively; Supplementary Table 5). This change may have been driven by a shift from a predominance of forests to more open vegetation, mainly the result of anthropogenic burning^36,58,59^. With the exception of relatively small (<1%) increases in Arecaceae (possibly *Cocos nucifera*) and Casuarinaceae pollen (possibly *Casuarina equisetifolia*) after human settlement of the islands, none of the most abundant taxa, either pre- or post-human settlement, appear to be obvious introductions or cultivars (see ref. ^44^ for discussion of introduced and cultivated plants).

### Site-specific factors

While human presence appears to be a key determinant of the magnitude of similarity among sites, island and site characteristics may also be important. For example, among 30 Eastern Atlantic Ocean islands, once colonized by humans, topographically complex islands maintained native vegetation cover suggesting that topography constrains human impacts on biodiversity^60^. Indeed, Lake Tagimaucia (Taveuni) and Lake Lanoto’o (Upolu) are two of the least floristically homogenized sites included in this analysis, and both are located at high elevations: 680 and 760 m above sea level (a.s.l.), respectively (Fig. 2). However, Waitetoke (Ahuahu) showed the opposite relationship, as it was one of the least floristically homogenized, but the site is located at 1 m a.s.l. The island’s close proximity to the North Island (Te Ika-a-Māui) of New Zealand may have maintained Ahuahu’s floristic diversity as propagules are continuously dispersed to Ahuahu from the mainland. Ahuahu’s floristic diversity may have contributed to its apparent dissimilarity to other sites. Other low-elevation sites, such as Avai’o’vuna Swamp (Pangaimotu), Ngofe Marsh (‘Uta Vava’u) and Finemui Swamp (Ha’afeva), located at 0 m, 4 m and 7 m a.s.l., respectively, were the most floristically homogenized, potentially because low-elevation and coastal areas are most exposed to intense anthropogenic ecosystem modifications. Island or site characteristics are likely complex predictors of an island’s susceptibility to becoming floristically homogenized.

It is important to highlight that pollen assemblages based on larger sample numbers may include more rare taxa, which may in turn make them appear to be dissimilar from other sites. To some extent, the use of Bray–Curtis similarity, which tends to underestimate the effects of rare taxa, may have removed some of the impacts of differing sample numbers. Indeed, the sites with the highest mean pollen and spore counts were Bonatoa Bog (Viti Levu) and St. Louis Lac (Grande Terre) (Supplementary Table 3), and these were neither the most nor the least floristically homogenous (Figs. 1 and 2). In addition, the trend towards floristic homogenization was also apparent when samples with low pollen counts (<300) were excluded from the analysis (Supplementary Fig. 15). This indicates that differences in sample size are unlikely to explain the observed large-scale patterns.

## Conclusions

This study provides an analysis showing that South Pacific island vegetation has become more homogenous over the past 5,000 years. Some narratives point to biotic homogenization being a largely contemporary issue exacerbated by increased commerce between islands^61^, which in turn increases dispersal possibilities for plant taxa. Our analysis indicates, however, that initial human settlement was likely a major driver of floristic homogenization. Future trends of floristic similarity will depend on the levels of continued human ecosystem modification, rates of non-native introductions and rates of extinctions and extirpations. This study highlights the need for long, standardized palaeoecological records that can be integrated with modern ecological observations to fully understand and effectively manage modern island ecosystems.

## Methodology

### Study sites

This study focuses on islands in the tropical, sub-tropical and warm–temperate Southwest Pacific Ocean and includes the more continental originating islands west of the Andesite Line^62^ (*n* = 12) and the strictly oceanic islands to the east (*n* = 3) within the biogeographical realm of Remote Oceania (Fig. 1). The main sources of precipitation are the Intertropical Convergence Zone and South Pacific Convergence Zone^63^. The island of Ahuahu (Great Mercury), which is offshore of New Zealand’s North Island (refs. ^64,65^), experiences a warm temperate climate. New Caledonia, Fiji and Vanuatu, located in the tropical Southwest Pacific geographic area, have floras similar to those of Australia and Papua New Guinea^66^. The more remote islands to the east are characterized by an Indo-Pacific flora, which includes species from the Americas, New Zealand and the sub-Antarctic^66^. Throughout the Pacific, plant diversity decreases from west to east, but larger and older islands tend to be more biodiverse^67^. The island areas included in this study range from Grande Terre, New Caledonia (1,890,000 ha) to Foa, Tonga (135 ha), and the islands’ geologies include composites, volcanics and limestone (Supplementary Table 1).

The arrival of oceanic voyagers in the Pacific islands represents the last wave of human migration into unoccupied lands (see Supplementary Table 1 and references therein). Archaeological evidence suggests that the first human migrations to Remote Oceania were by the Lapita^68^. Linguistic studies indicate Lapita origins to have been somewhere between Taiwan and the Bismarck Archipelago^69^, and genetic studies support this inference (see refs. ^70–73^ for discussion). After a pause of ∼1,700 years (∼2,700 to 1,000 cal years BP), Polynesians, descendants of the Lapita, undertook the last great migration east into the Pacific (for example, refs. ^63,74^).

### Acquisition of pollen datasets

Pollen records from 15 sites (Supplementary Table 1) are included in this analysis. These sites were chosen because together they represent both early (∼3,000 cal years BP) and late (∼700 cal years BP) human colonization and provide pollen records at least 3,300 years long, allowing comparisons between pre- and post-settlement pollen assemblages (Supplementary Fig. 6). In addition, they form an elevational gradient from 0 m to 760 m a.s.l. Sites at different elevations allow us to investigate trends from coastal regions, where humans have typically settled, to upland areas that may have escaped some of the biotic impacts of human disturbances^60,75^.

Five datasets were accessed via the Neotoma Paleoecology Database^76^ using the neotoma2 R package (version 0.0.0.9)^77^. These include Anouwe Swamp (Aneityum)^78^, Bonatoa Bog (Viti Levu), Lake Tagimaucia (Taveuni)^79^, Tukou Marsh (Rapa) and Waitetoke (Ahuahu/ Great Mercury)^65^. We excluded the pollen data from Lake Emeric, New Caledonia^80^, which also can be accessed via the Neotoma Paleoecology Database, due to low pollen and spore counts (∼60 per sample for the past 5,000 years). The following pollen datasets were acquired from published papers: Ngofe Marsh (‘Uta Vava’u), Lotofoa Swamp (Foa), Finemui Swamp (Ha’afeva), Avai’o’vuna Swamp (Pangaimotu)^35,58^, Lake Lanoto’o (Upolu)^75^, Plum Swamp (Grande Terre)^81^, St. Louis Lac (Grande Terre)^82^, Yacata (Yacata Island) and Volivoli (Viti Levu)^79^. One pollen record from Rano Aroi (Rapa Nui) was digitized manually using ImageJ software (version 1.53e)^83^.

### Age–depth models

The fossil pollen datasets from Anouwe Swamp, Avai’o’vuna Swamp, Bonatoa Bog, Finemui Swamp, Lake Lanoto’o, Lake Tagimaucia, Lotofoa Swamp, Ngofe Marsh, Tukou Marsh and Waitetoke already included calibrated ages (see Supplementary Table 2 for details of calibration curves). The age–depth models for Plum Swamp, Rano Aroi, St. Louis Lac, Volivoli and Yacata were recalibrated using the SHCal20 calibration curve using rbacon (version 3.0.0)^84,85^ (Supplementary Figs. 1–5). We rejected one date (50 cm depth) from the Volivoli age–depth model (Supplementary Fig. 3) as it was based on freshwater shells and the original publication indicated that it likely contained old carbon^79^.

### Standardization of pollen datasets

We matched pollen and spore names to accepted plant names from Plants of the World Online^86^ using the R package taxize (version 0.9.100)^87^. As pollen identification is often limited taxonomically^88^, we use two methods to improve the thoroughness of our approach. Both approaches, standardization-1 and standardization-2, removed the categories of indeterminate pollen and spores and taxa that indicated uncertainty at levels higher than family. Both approaches replaced synonyms with the most recently accepted names. Likewise, pollen and spore names with multiple matches were aggregated up to one taxonomic level if they were members of the same genus or family; for example, *Macaranga*/*Mallotus* was updated to Euphorbiaceae. Standardization-1 preserves the lowest level of identification (for example, *Ipomoea* cf. *batatas* was updated to *Ipomoea batatas*), while standardization-2 uses the higher level of identification (for example, *Ipomoea*). Standardization-1 resulted in 383 taxa (family = 81, genus = 253, species = 49), and standardization-2 resulted in 361 taxa (family = 102, genus = 218, species = 41).

### Statistical analyses

As methods of pollen sampling differ over time and among sites (Supplementary Table 3), we rarefied the original pollen counts (excluding unknown types) before standardization using the R package vegan (version 2.6-4) to allow for temporal and between-site comparisons^89,90^ (Supplementary Figs. 7 and 8). Rarefaction was not possible for the three pollen records from which only pollen percentage data were available, specifically, digitized records Lotofoa Swamp and Finemui Swamp and the Rano Aroi dataset digitized for this analysis.

To allow for temporal comparisons within both the standardization-1 and standardization-2 datasets, pollen samples from each site were placed within ten 500 year intervals, and pollen data within these intervals were averaged to obtain the mean values, resulting in 132 averaged pollen assemblages for analysis. Before averaging, pollen data within the earliest eight 500 year intervals contained ∼30 samples from all sites, and the two most recent time intervals contained ∼45 samples from all sites (Supplementary Table 4 and Supplementary Figs. 9 and 10). Pollen counts were transformed to percentages before further statistical analyses. We defined unique pairings among the 15 sites, thus comparing each site with all others resulting in 105 unique pairs (excluding comparisons within a given site). We then calculated the Bray–Curtis dissimilarity index^33^ for each pairing within each time interval (thereby creating 810 individual pairwise comparisons). Bray–Curtis dissimilarity was selected as it takes abundance into account, whereas other methods (for example, the Jaccard dissimilarity index) are based on presence/absence data. This method also excludes joint absences, and thus sites with the same missing taxa are not deemed more similar^91^. Bray–Curtis dissimilarity values (0–1) were inverted so the values represent taxonomic similarity, with 1 being the most similar (100%) and 0 being the least (0%).

Linear models identify the slope coefficients of trends in similarity over time, with negative trends (<0) representing homogenization and positive trends (>0) representing the opposite, differentiation. The slopes thus indicate how often a site’s pollen assemblages tend to become more floristically similar (homogenization) or less floristically similar (differentiation) to those of the other sites through time. Each slope coefficient is derived from a minimum of four data points. The individual pairwise comparisons can be from a period when neither, one or both of the islands were settled by people. Two site pairings compared time frames when both islands were settled, 81 pairings compared sites with two of the settled statuses (neither, one or both), and 22 pairings compared sites from all three of the settled statuses. To assess the effect of human settlement on floristic similarity, pairwise comparisons were defined according to whether both sites were settled by people, just one of the pair was settled or if neither was settled during a given time interval.

Smoothing spline models were created using the npreg package (version 1.0-9)^92^ to plot overall changes in taxonomic similarity over time in the standardization-1 and in the standardization-2 datasets. See the supplementary information (Supplementary Figs. 14–16) for results of the sensitivity analyses based on pairwise comparisons between taxa within the same rank (for example, genus with genus) (Supplementary Fig. 14), on pollen samples with counts <300 that are excluded from the analysis (Supplementary Fig. 15) and on the pollen datasets excluding abundant taxa (Cyperaceae and Poaceae) (Supplementary Fig. 16).

### Reporting summary

Further information on research design is available in the Nature Portfolio Reporting Summary linked to this article.

### Supplementary information

Supplementary Information This file contains supplementary details of additional sensitivity analyses, Supplementary Tables 1–5 and Figs. 1–16.

Reporting Summary

## Data Availability

All data are available via Github at https://github.com/nastrandberg/Biotic_homogenisation and figshare at 10.6084/m9.figshare.22794191.

## Code availability

All code is available via Github at https://github.com/nastrandberg/Biotic_homogenisation and figshare at 10.6084/m9.figshare.22794191.
