# Supplementary material for: Floristic homogenization of South Pacific islands commenced with human arrival
Source: Nat Ecol Evol. 2024 Jan 15;8(3):511–8. doi: 10.1038/s41559-023-02306-3 (PMC10927543; doi:10.1038/s41559-023-02306-3)
Supplement: Supplementary file 1 — This file contains supplementary details of additional sensitivity analyses, Supplementary Tables 1–5 and Figs. 1–16. [file 41559_2023_2306_MOESM1_ESM.pdf]

nature ecology & evolution

Article https://doi.org/10.1038/s41559-023-02306-3

Floristic homogenization of South Pacific islands commenced with human arrival

In the format provided by the authors and unedited

Sediment compaction and pollen richness

To investigate whether pollen richness was greater in more compacted sediments, for example, those from lower down in the sediment stratigraphy of cores, we plotted pollen richness and rarefied pollen richness against accumulation time (years) per 1 cm. This was done for three sites where pollen sampling thickness and age-depth model outputs were available, St. Louis Lac, Ngofe Marsh, and Lake Lanoto'o (Fig. S8). The accumulation times for 1 cm of sediment were based on outputs from the *rbacon* age-depth models. Fig. S8 shows that there was no obvious relationship between either pollen richness or rarefied pollen richness and sediment accumulation time.

Sensitivity analysis

To test the robustness of the trend towards floristic homogenization we used two additional approaches in combination with standardization-1 and standardization-2. In the first approach we compared taxa within the same rank within the pairwise similarity comparisons, e.g., family level taxa with family level taxa. Both standardization-1 and standardization-2 show a linear increase in similarity over the last 5000 years (Fig. S14). The similarity of standardization-1 was 0.06 at 5000 cal. years BP and increased to 0.12 towards the present day. The similarity of standardization-2 was 0.07 at 5000 cal. years BP and increased to 0.14 towards the present day (Fig. S14). These findings are consistent with the results where taxa of all taxonomic ranks are compared (Fig. 3A).

In the second approach we excluded samples with pollen and spore counts <300 to test how low pollen counts potentially impact the trend. Data from digitized sites i.e., Rano Aroi, Finemui Swamp, and Lotofoa Swamp where only percentage data was available were removed. All data from Anouwe Swamp, Lake Lanoto'o, and Tukou Marsh were also removed since these all had pollen and spore sums <300 after standardization. Both standardization approaches had a similarity of ~0.06 5000 years ago and both approaches also had a similarity value of ~0.12 towards the present day (Fig. S15). Both approaches had enhanced similarity between 3000 and 1000 cal. years BP, standardization-2 showed increased values of 0.16 during this time whereas standardization-1 reached maximum similarity values of 0.11 (Fig. S15). The trends of increasing similarity over the last 5000 years are also consistent with the analysis where pollen and spore samples with all counts, including those <300, are included (Fig. 3A).

Impact of abundant pollen types on the similarity analysis

We tested the effect of excluding the two most abundant taxa after human settlement of the islands (Cyperaceae which had a mean abundance of ~17% and Poaceae which had a mean abundance of 10% after human arrival), to investigate their impact on the overall similarity scores (see table S5 for list of the most abundant taxa before and after human colonization).

When Cyperaceae was excluded from the analysis, and the remaining taxa were rescaled to 100%, the overall similarity increased during the last 5000 years for both the standardization-1 and standardization-2 approaches (Fig. S16A). At 5000 cal. years BP similarity was 0.07 and 0.08 for standardization approaches 1 and 2 respectively. For the most recent time interval, similarity had increased to 0.12 and 0.14 for standardization-1 and standardization-2 respectively.

When Poaceae was excluded from the analysis, and the remaining taxa were rescaled to 100%, both standardization approaches also showed increases in similarity over the last 5000 years (Fig. S16B). The similarity scores at 5000 cal. years BP were 0.07 and 0.08 for standardization-1 and standardization-2 respectively. Similarity increased to 0.11 and 0.16 for standardization-1 and standardization-2 respectively by the most recent time interval. These analyses indicate that neither Cyperaceae nor Poaceae are the sole drivers of the homogenization trend but that both contribute to higher similarity scores in the more recent time intervals.

Table S1. Paleoecological records included in this study. Island type is from Nunn et al. (2016) and classifications with the * symbol are from Hayward (1976). Max. elev.= maximum island elevation (m) from Nunn et al. (2016) and values with the * symbol are from Google Earth Pro (2023). Distance= distance from nearest mainland (km) from Weigelt et al. (2013) and for values with the * symbol from Google Earth Pro (2023).

Site name	Site ID (this study)	Neotoma Site ID	Latitude	Longitude	Site elevation (m.a.s.l)	Country	Island name	Approximate arrival of humans (Cal. years BP)	Island area (ha) Nunn et al. (2016) *or Google Earth Pro (2023)	Island type	Max. elev. (m)	Distance (km)	Minimum age Cal year BP	Maximum age Cal years BP	Type	Reference/ Pollen analyst
**St. Louis Lac**	1	28240	-22.23278	166.55	5	New Caledonia	Grande Terre	~3000 (Sand, 1997)	1891479.34	Continental island	1628*	1353.55	592	6688	Wetland	Stevenson and Dodson (1995) and Stevenson (2004)
**Plum Swamp**	2	NA	-22.261461	166.626675	10	New Caledonia	Grande Terre	~3000 (Sand, 1997)	1891479.34	Continental island	1628*	1353.55	124	23,020	Wetland	Stevenson et al. (2001)
**Anouwe Swamp**	3	28318	-20.23932	169.82227	4	Vanuatu	Aneityum	3000 (Petchey et al., 2014)	17928.56	Volcanic high island	852	1816.91	1419	16,087	Wetland	Hope and Spriggs (1982)
**Waitetoke**	4	23921	-36.61015	175.78865	1	New Zealand	Ahuahu/ Great Mercury	700 (Wilmshurst et al., 2011)	1872*	Volcanic*	231*	6*	-58	5370	Wetland	Holdaway et al. (2019) and Prebble et al. (2019)
**Volivoli**	5	NA	-17.310000	178.170000	2	Fiji	Viti Levu	2900 (Anderson and Clark, 1999)	1182706	Composite high island	1323	2706.89	0	4873	Lagoon	Hope et al. (2009)
**Bonatoa Bog**	6	9979	-18.0667	178.5333	4	Fiji	Viti Levu	2900 (Anderson and Clark, 1999)	1182706	Composite high island	1323	2706.89	-34	4075	Wetland	Hope et al. (2009)
**Lake Tagimaucia**	7	27226	-16.8217	-179.939	680	Fiji	Taveuni	2900 (Anderson and Clark, 1999)	52728.56	Volcanic high island	1241	2829.38	83	17,062	Lake	Hope et al. (2009) and Southern (1986)
**Yacata**	8	NA	-17.258464	-179.510585	2	Fiji	Yacata	2900 (Anderson and	943.95	Composite high island	256	~2922.1	0	4939	Lake	Hope et al. (2009)

								Clark, 1999)								
**Finemui Swamp**	9	NA	-19.9489	-174.712	7	Tonga	Ha'afeva	2765 (Burley et al., 2015)	135.45	Limestone low island	14	3340.86	-69	5874	Wetland	Flenley et al. (1999)
**Lotofoa Swamp**	10	NA	-19.7467	-174.308	3	Tonga	Foa	2765 (Burley et al., 2015)	1571.78	Limestone low island	24	3385.72	-49	7008	Wetland	Flenley et al. (1999)
**Ngofe Marsh**	11	NA	-18.6647	-174.044	4	Tonga	‘Uta Vava'u	2780 (Burley et al., 2015)	12719.52	Limestone high island	88	3468.1	-67	7693	Wetland	Strandberg et al. (2023)
**Avai’o’vuna Swamp**	12	NA	-18.697111	-173.988747	0	Tonga	Uta Vava'u/ Pangaimotu	2780 (Burley et al., 2015)	12719.52	Limestone low island	8	3468.1	-47	4987	Wetland	Fall (2005)
**Lake Lanoto'o**	13	NA	-13.9093	-171.828	760	Samoa	Upolu	2800 (Leach and Green, 1989)	121546.4	Volcanic high island	1100	3887.14	545	10,004	Lake	Gosling et al. (2020)
**Tukou Marsh**	14	23925	-27.604	-144.352	3	French Polynesia	Rapa	700 (Kennett et al., 2006)	5413.68	Volcanic high island	650	6009.78	-32	8250	Wetland	Prebble et al. (2019)
**Rano Aroi**	15	NA	-27.1865	-109.435	430	Chile	Rapa Nui/ Easter Island	700 (Hunt and Lipo, 2006)	21350.09	Volcanic high island	507	3523.41	-27	41,506	Wetland	Flenley et al. (1991)

Table S2 Chronologies and pollen sampling thickness.

Site name	Materials dated	Dating technique	Calibration curve	Software used	Pollen sampling thickness/ volume	Comments
**Plum Swamp**	Bulk material	Conventional beta-counting and AMS	Recalibrated using SHcal20	*rbacon* v3.1.0	1 cm	The top 150 cm of the core was removed from the analysis since it is affected by mining spoil.
**Lac St. Louis**	Bulk material	Conventional beta-counting	Recalibrated using SHcal20	*rbacon* v3.1.0	1 cm	
**Anouwe Swamp**	Bulk material and plant remains	Unknown (likely to be conventional beta-counting)	SHcal20	*rbacon*	5 cm	
**Waitetoke**	Plant macrofossils and charcoal	AMS	SHcal13	OXCAL v4.3	1 cm^3	
**Volivoli**	Fine sediments	Unknown (likely to be conventional beta-counting)	Recalibrated using SHcal20	*rbacon* v3.1.0	Unknown	As per the original publication, a marine correction of 300 years was applied to the mollusc date at 28 cm. A date at 50 cm was rejected as the original publication indicates that the freshwater shells may have incorporated older carbon.
**Bonatoa Bog**	Bulk material and organic material	1 AMS date. Others unknown (likely to be conventional beta-counting)	Unknown	*rbacon*	Unknown	
**Lake Tagimaucia**	Bulk material	Unknown (likely to be conventional beta-counting)	SHcal13	*rbacon*	1 cm^3	
**Yacata**	Acid insoluble shelly peat and organic fines	Unknown (likely to be conventional beta-counting)	Recalibrated using SHcal20	*rbacon* v3.1.0	Unknown	
**Finemui Swamp**	Bulk material	Unknown (likely to be conventional beta-counting)	Recalibrated using SHcal20	*rbacon* v3.1.0	Unknown	

**Lotofoa Swamp**	Bulk material	Unknown (likely to be conventional beta-counting)	Recalibrated using SHcal20	*rbacon* v3.1.0	Unknown
**Ngofe Marsh**	Plant macrofossils and bulk material	AMS	SHcal20	*rbacon* v3.1.0	$1cm^3$
**Avai'o'vuna Swamp**	Bulk material	Conventional beta-counting	Recalibrated using SHcal20	*rbacon* v3.1.0	Unknown
**Lake Lanoto'o**	Plant macrofossils and bulk material	^{210}Pb, ^{137}Cs and AMS ^{14}C dating	SHcal13	*rbacon* v2.2	$1cm^3$
**Tukou Marsh**	Plant macrofossils and pollen concentrate	AMS	SHcal13	OXCAL v4.3	$1\ cm^3$
**Rano Aroi**	Unknown	Unknown (likely to be conventional beta-counting)	Recalibrated using SH20	*rbacon* v3.1.0	Unknown

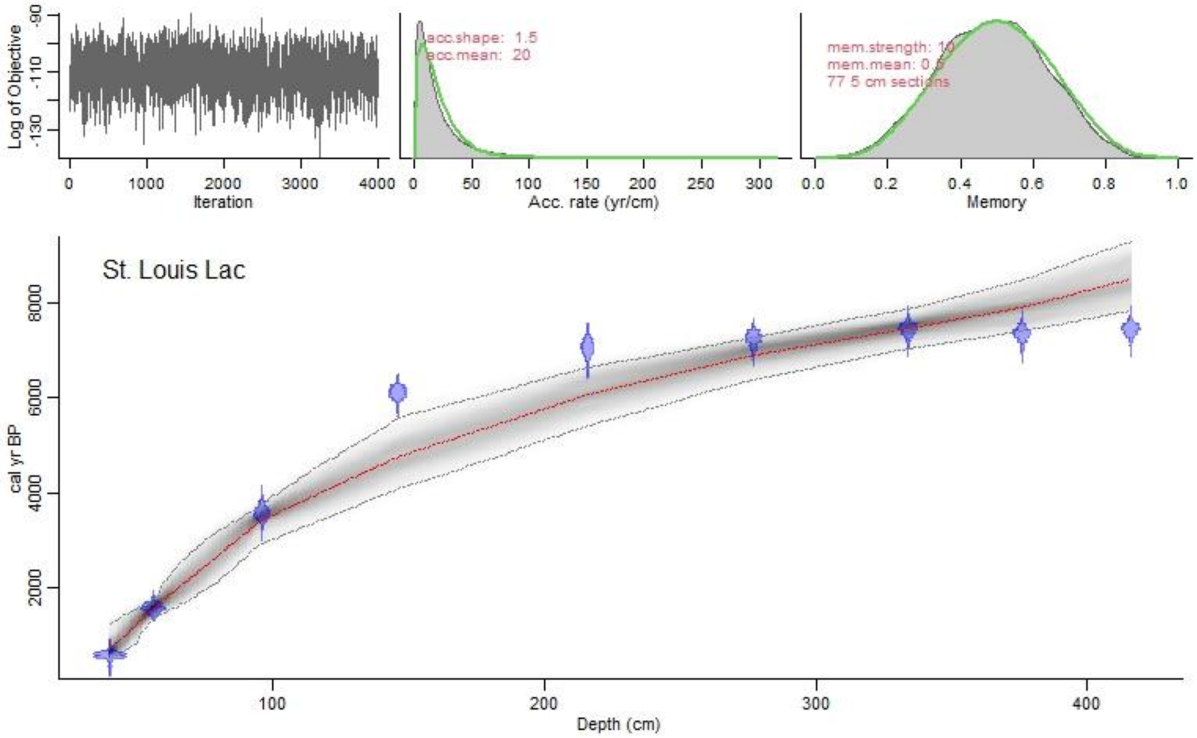

Figure S1. Age depth model for St. Louis Lac updated from Stevenson and Dodson (1995). Radiocarbon dates were recalibrated using the SHCal20 calibration curve using *rbacon* (Blaauw and Christen, 2011; Hogg et al., 2020). The gray shaded area represents a confidence interval of 95%.

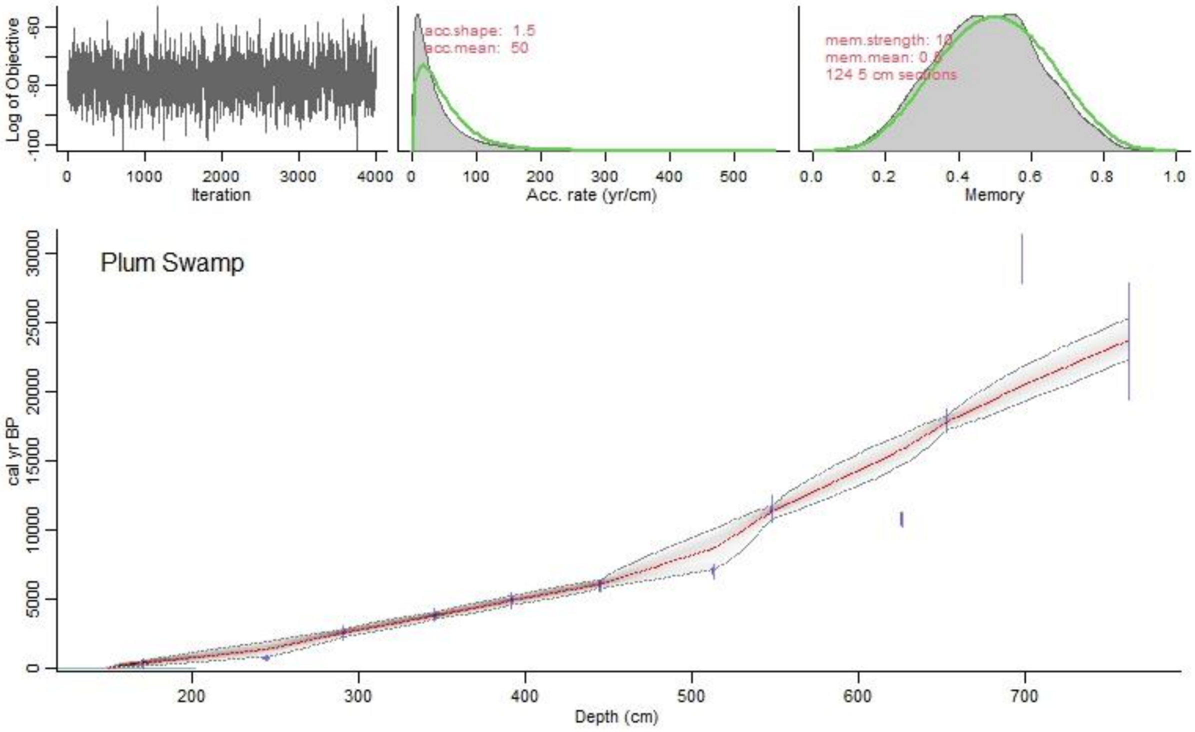

Figure S2. Age depth model for Plum Swamp updated from Stevenson et al. (2001). Radiocarbon dates were recalibrated using the SHCal20 calibration curve using *rbacon* (Blaauw and Christen, 2011; Hogg et al., 2020). The gray shaded area represents a confidence interval of 95%.

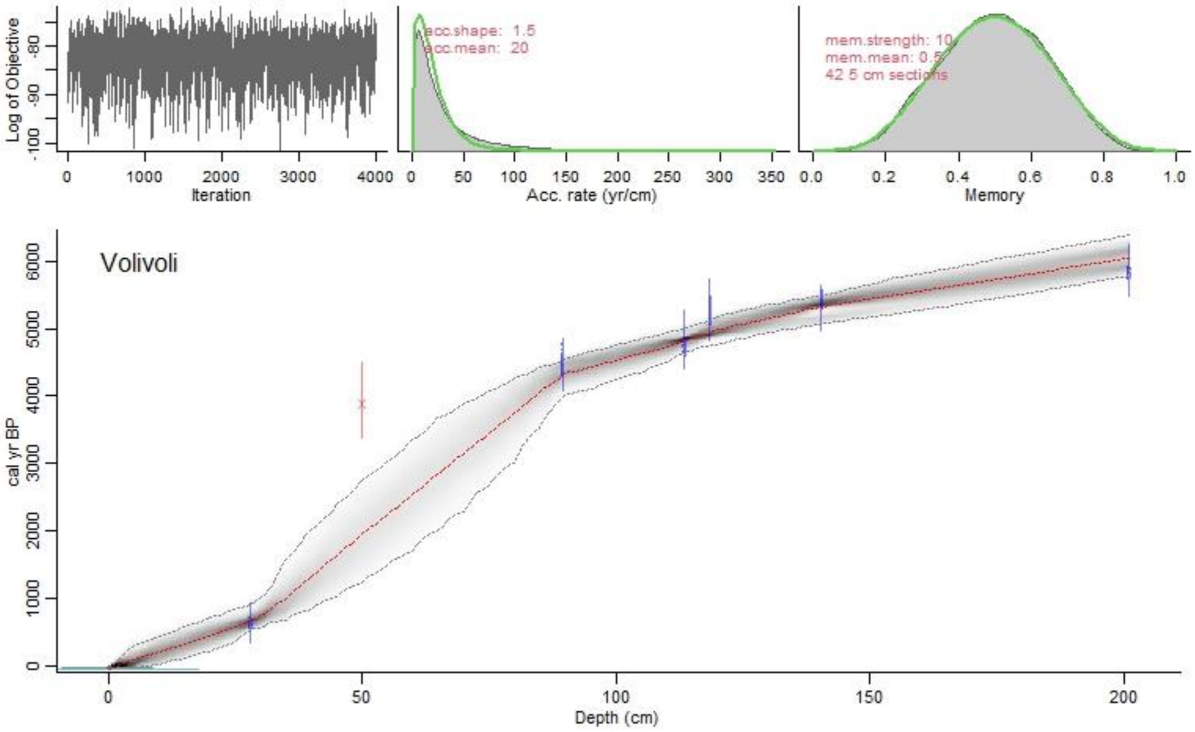

Figure S3. Age depth model for Volivoli updated from Hope et al. (2009). Radiocarbon dates were recalibrated using the SHCal20 calibration curve using *rbacon* (Blaauw and Christen, 2011; Hogg et al., 2020). The gray shaded area represents a confidence interval of 95%.

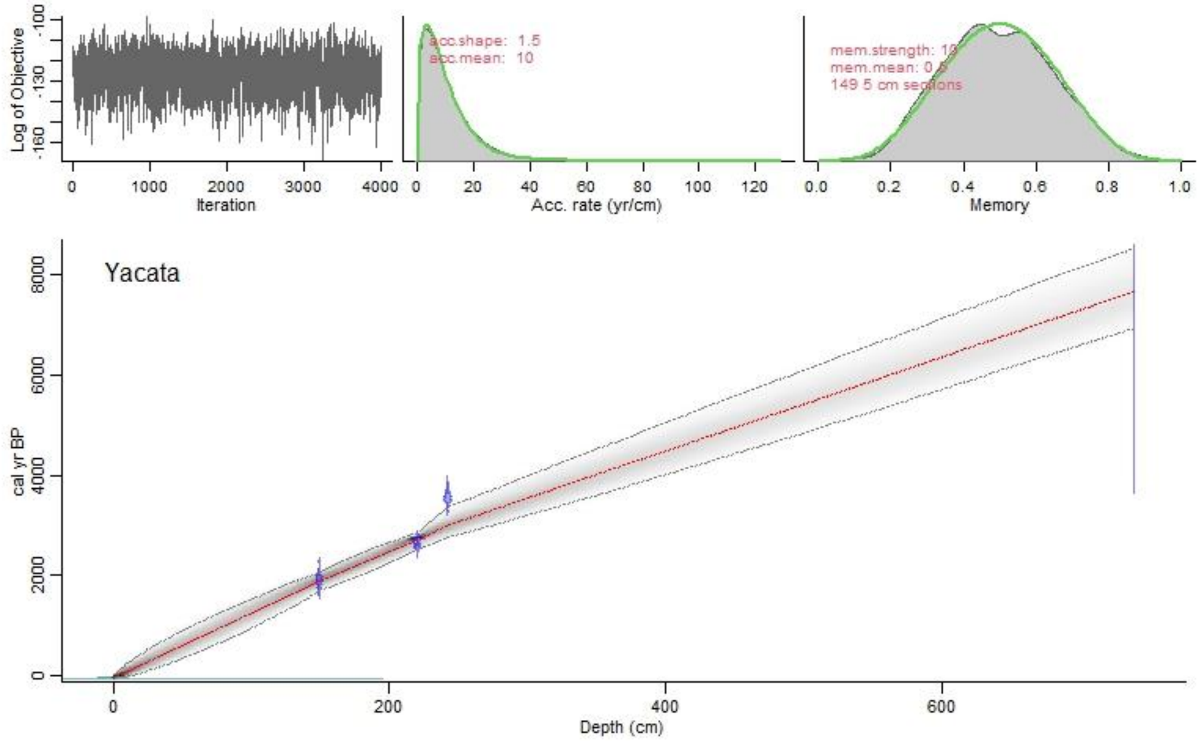

Figure S4. Age depth model for Yacata updated from Hope et al. (2009). Radiocarbon dates were recalibrated using the SHCal20 calibration curve using *rbacon* (Blaauw and Christen, 2011; Hogg et al., 2020). The gray shaded area represents a confidence interval of 95%.

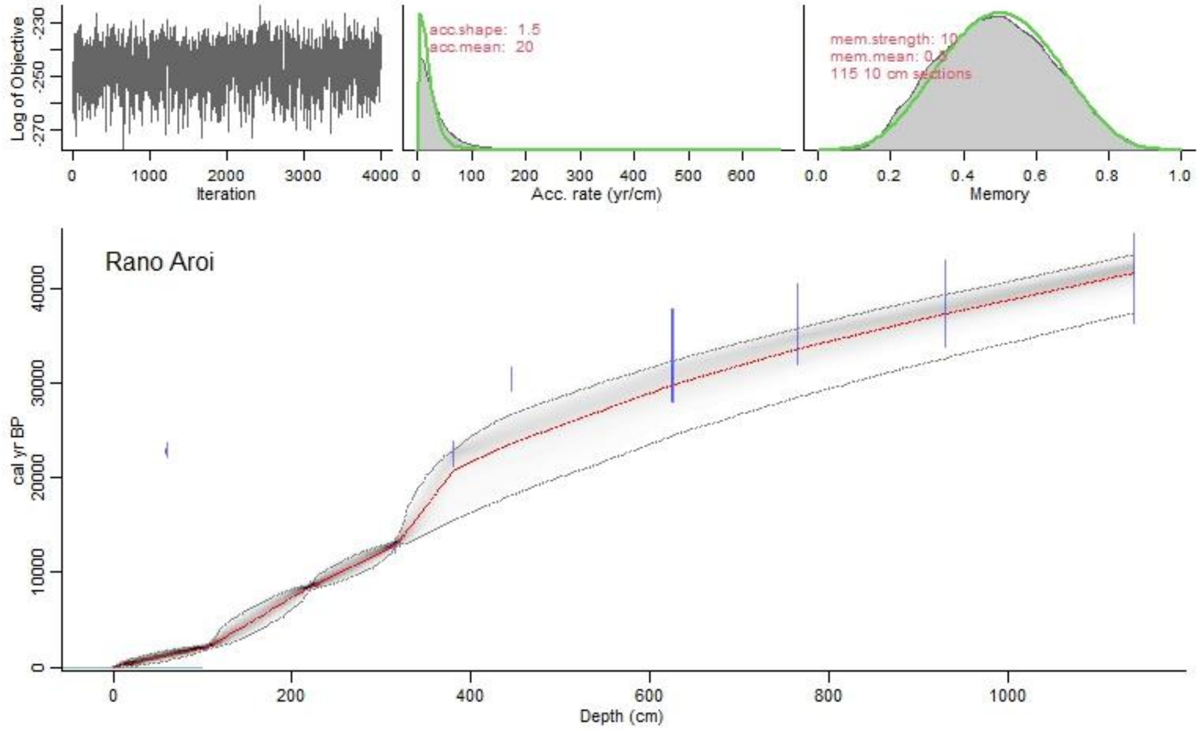

Figure S5. Rano Aroi age-depth model updated from Flenley et al. (1999). Radiocarbon dates were recalibrated using the SHCal20 calibration curve using *rbacon* (Blaauw and Christen, 2011; Hogg et al., 2020). The gray shaded area represents a confidence interval of 95%.

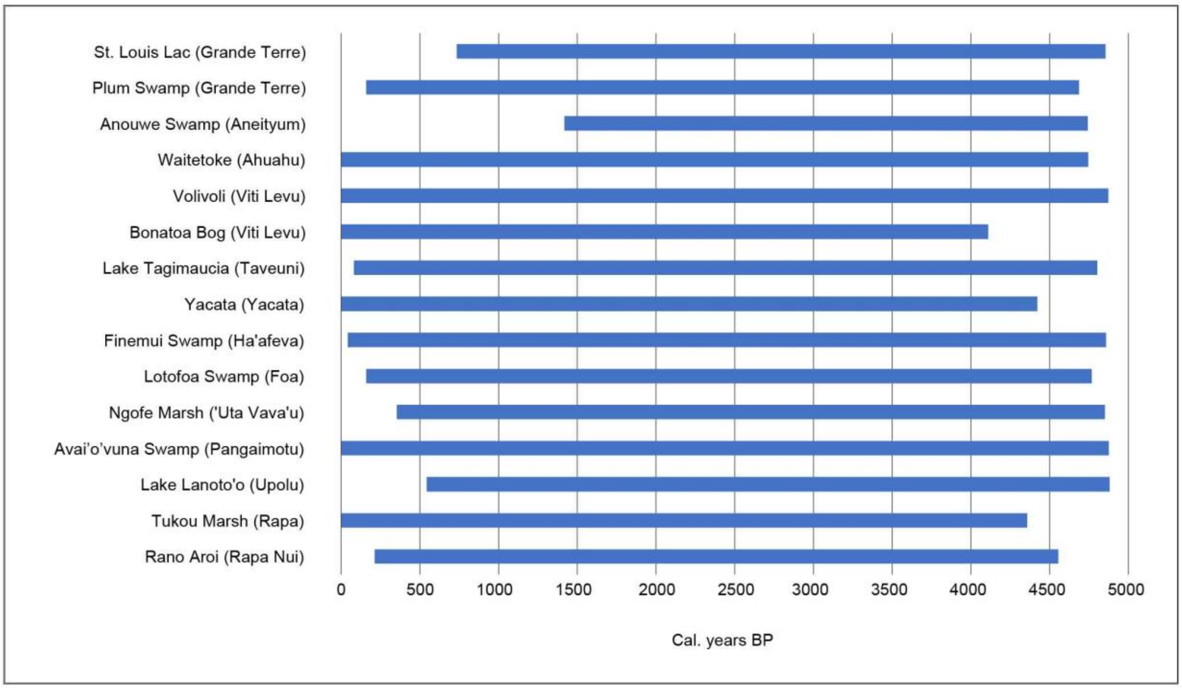

Figure S6. The length of pollen records included in this study with pollen assemblages older than 5000 cal. years BP removed.

Table S3. Descriptions of the original pollen data excluding indeterminate types without type codes or damaged types.

**Site name**	**Pollen data format (prior to rescaling to %)**	**Mean pollen & spore sum**	**Minimum pollen & spore sum**	**Total number of taxa**	**Mean rarefied richness**	**Reference/ Pollen analyst**
Lac St. Louis	Count	780	70	155	16	Stevenson (2004)
Plum Swamp	Count	546	14	140	7	Stevenson et al. (2001)
Anouwe Swamp	Count	164	10	55	6	Hope and Spriggs (1982)
Waitetoke	Count	385	244	105	25	Holdaway et al. (2019) and Prebble et al. (2019)
Volivoli	Count	285	83	71	18	Hope et al. (2009)
Bonatoa Bog	Count	1092	311	98	27	Hope et al. (2009)
Lake Tagimaucia	Count	479	238	132	37	Hope et al. (2009) and Southern (1986)
Yacata	Count	373	76	57	9	Hope et al. (2009)
Finemui Swamp	Percentage	NA	Normally 200 but occasionally fewer	38	NA	Flenley et al. (1999)
Lotofoa Swamp	Percentage	NA	Normally 200 but occasionally fewer	34	NA	Flenley et al. (1999)
Ngofe Marsh	Count	251	54	74	11	Strandberg et al. (2023)
Avai'o'vuna Swamp	Count	403	81	82	18	Fall (2005)
Lake Lanoto'o	Count	314	279	43	22	Gosling et al. (2020)
Tukou Marsh	Count	298	249	53	18	Prebble et al. (2019)
Rano Aroi	Percentage	NA	200 terrestrial pollen in most cases	17	NA	Flenley et al. (1991)

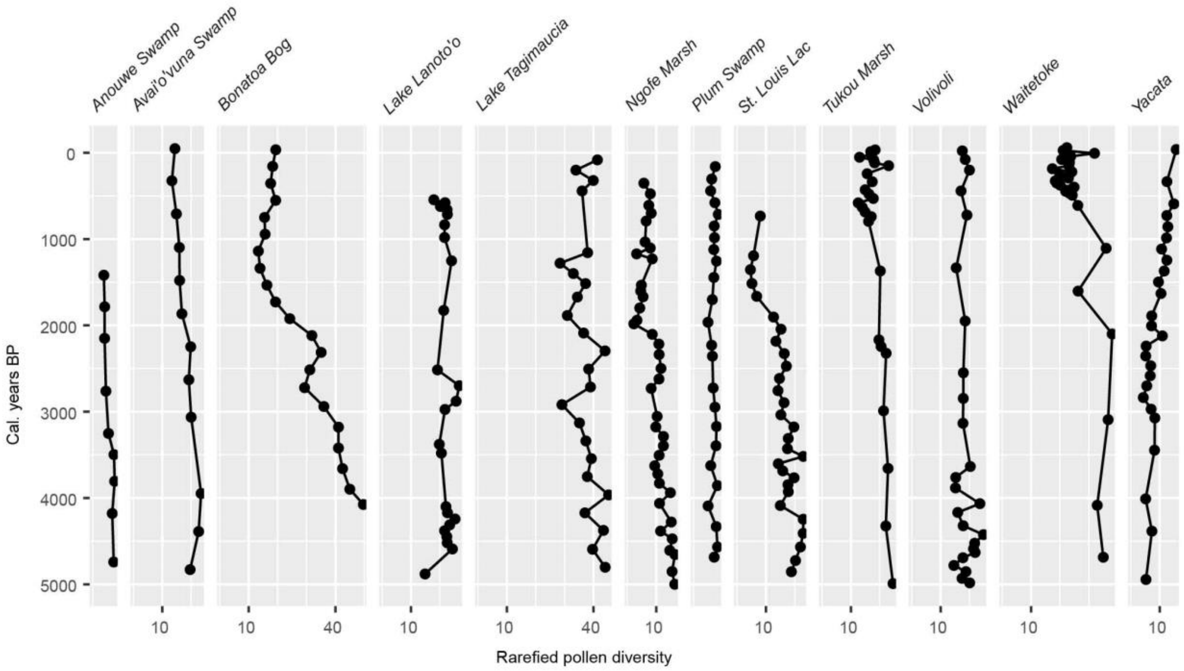

Figure S7. Rarefied pollen and spore diversity for the last 5000 years.

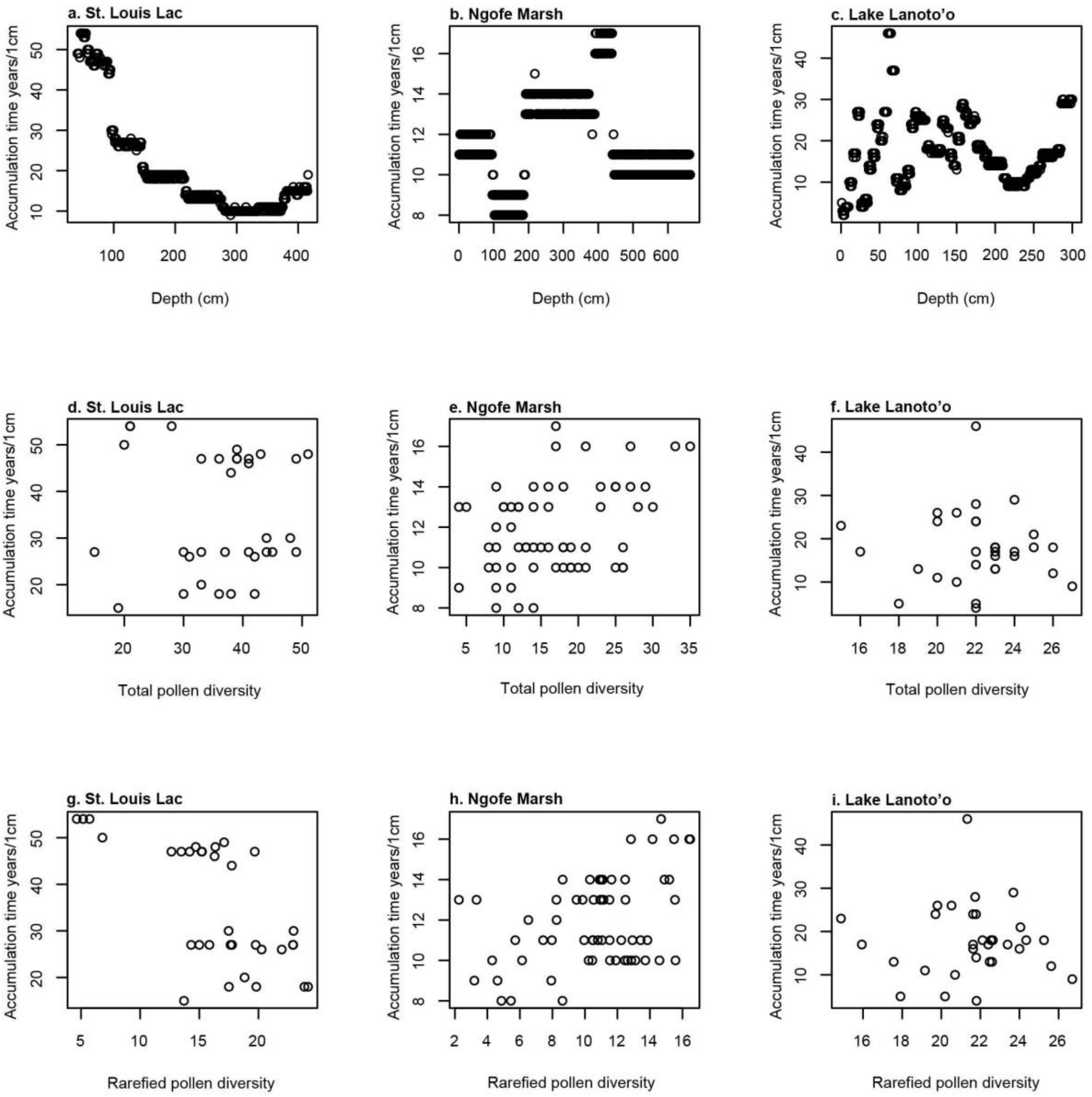

Figure S8. Sediment accumulation times for three selected sites. The sediment accumulation times (years) per 1 cm (derived from the age-depth model) are plotted against depth (cm), total pollen diversity, and rarefied pollen diversity for St. Louis Lac, Ngofe Marsh, and Lake Lanoto'o.

Table S4 the number of pollen assemblages contained within each time interval.

Mean interval time (Cal. years BP)	Anouwe Swamp (Aneityum)	Rano Aroi (Rapa Nui)	Avai'o'vuna Swamp (Pangaimotu)	Bonatoa Bog (Viti Levu)	Finemui Swamp (Ha'afeva)	Lake Lanot o'o	Lotofoa Swamp (Foa)	St. Louis Lac (Grande Terre)	Ngofe Marsh ('Uta Vava'u)	Plum Swamp (Grande Terre)	Lake Tagimaucia (Taveuni)	Tukou Marsh (Rapa)	Volivoli (Viti Levu)	Waiteto ke (Ahuah u)	Yacata (Yacata Island)
150	0	2	2	3	3	0	1	0	1	2	3	9	3	15	2
650	0	2	1	2	3	6	2	1	4	4	1	8	2	5	3
1150	0	1	1	3	3	2	2	2	4	3	3	1	1	1	4
1650	2	2	2	2	3	1	1	2	4	2	3	0	0	1	3
2150	1	0	1	3	2	0	2	4	5	3	2	3	1	1	4
2650	1	2	1	2	1	3	1	4	3	1	2	0	2	0	4
3150	1	1	1	2	2	2	1	3	4	3	3	1	1	1	2
3650	2	0	0	3	2	1	3	6	4	2	2	1	3	0	1
4150	1	0	2	1	2	5	1	3	4	2	3	1	3	1	2
4650	1	1	1	0	2	4	2	4	4	2	2	0	7	1	0

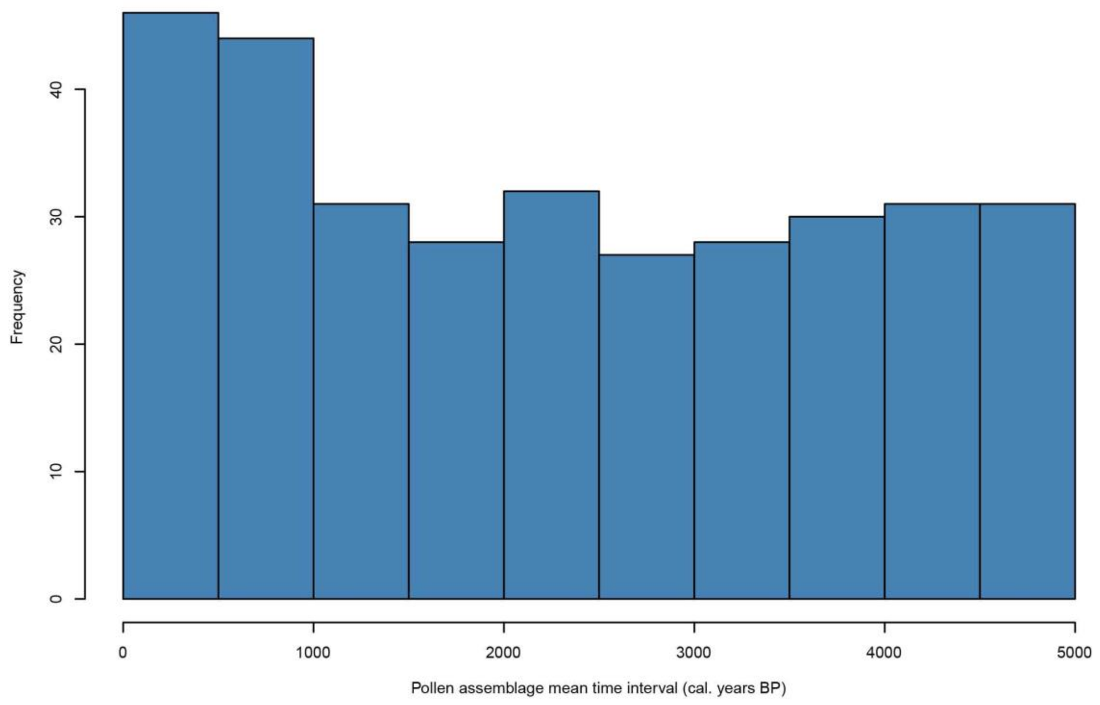

Figure S9. The number of pollen samples within each 500-year time interval.

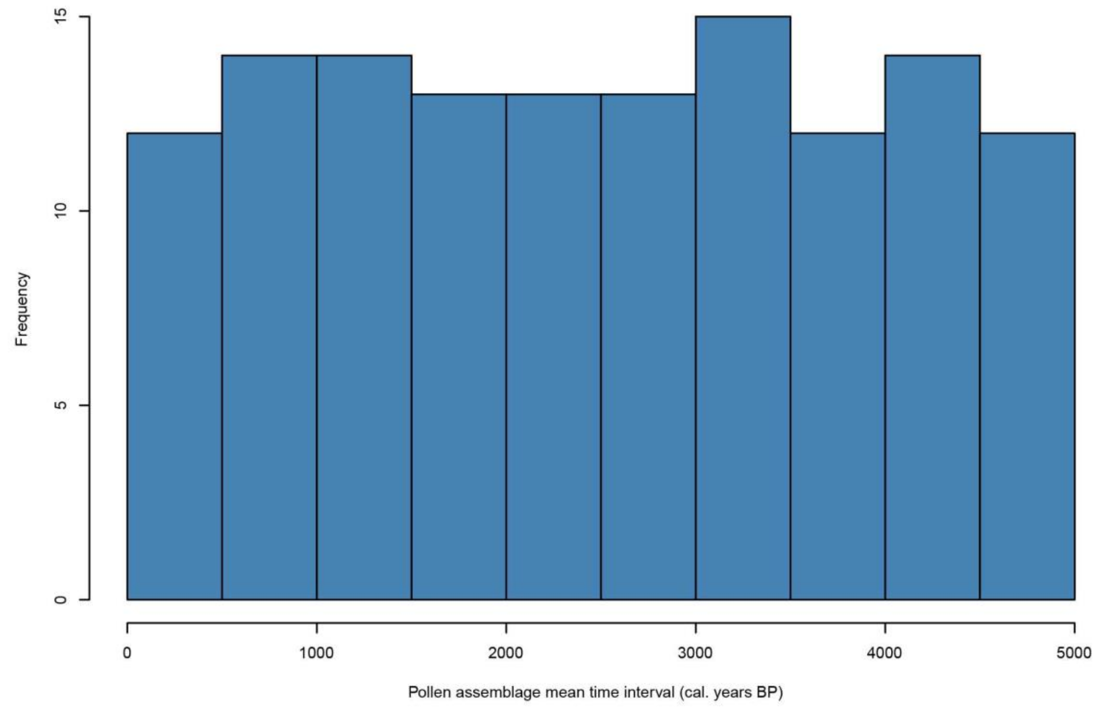

Figure S10. Number of 500-year time intervals for all sites.

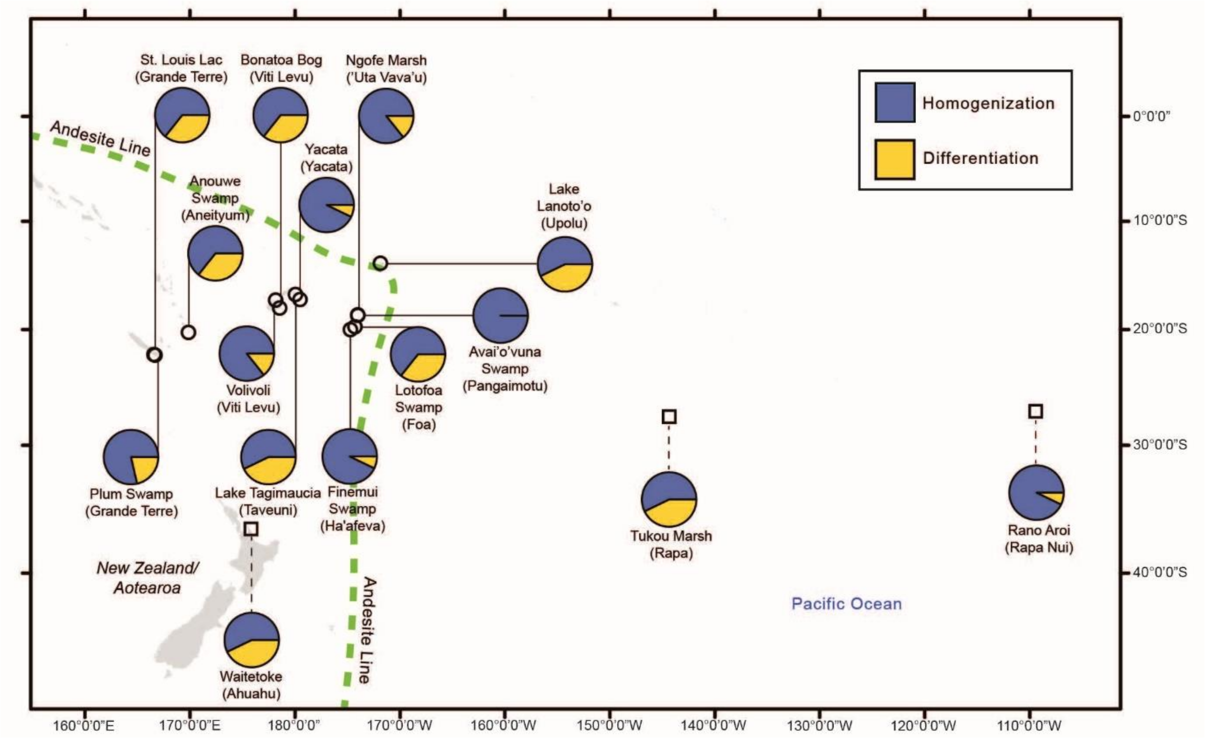

Figure S11. Proportions for each site of Bray-Curtis similarity homogenizing trends (<0 slope coefficients) in blue and differentiating trends (>0 slope coefficients) in yellow over time based on standardization-2. The Andesite Line is shown as a green dashed line. Circles with solid leader lines indicate sites settled ~3000 cal. years BP and squares with dashed leader lines indicate sites settled ~700 cal. years BP. Island names follow site names in parentheses. X- and Y- axis represent longitude and latitude, respectively.

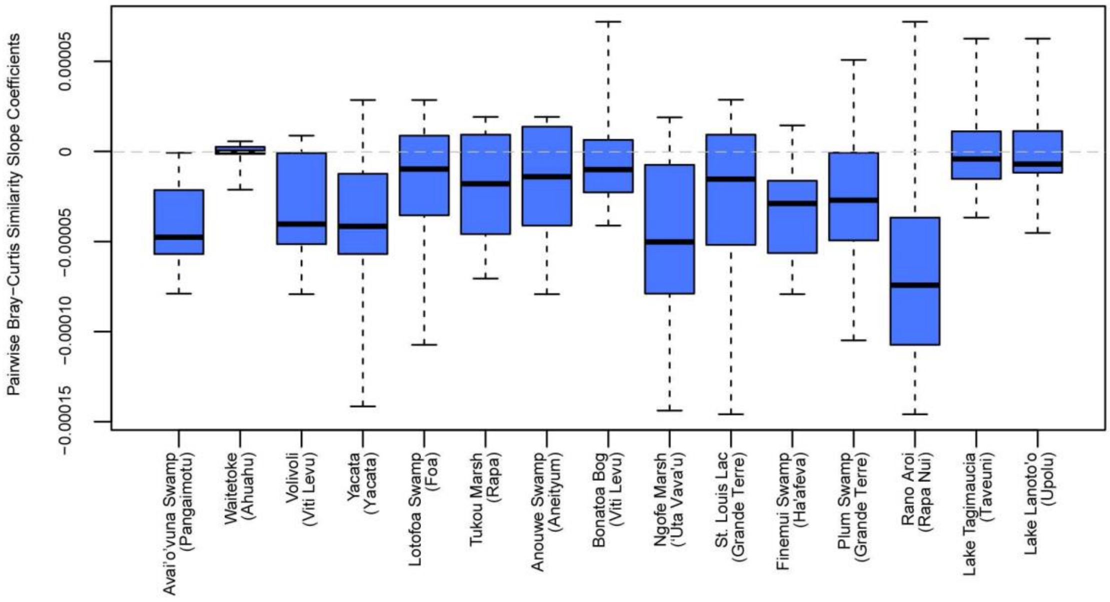

Figure S12. Pairwise Bray-Curtis floristic similarity trends for each site. The direction and steepness of the floristic similarity trends based on standardization-2 between sites (*n*= 14 site comparisons since we exclude comparisons within a given site) based on pairwise Bray-Curtis similarity slope coefficients. Sites are organized by elevation with the lowest (sea

level) on the left to the highest on the right (760 m.a.s.l). Data points above the gray horizontal dashed line are differentiating trends and below this line are homogenizing trends. The black horizonal lines indicate the medians of the data for each site and the blue bars encompass the first and third quantiles of the data. Whiskers extend to the maximum and minimum of the data.

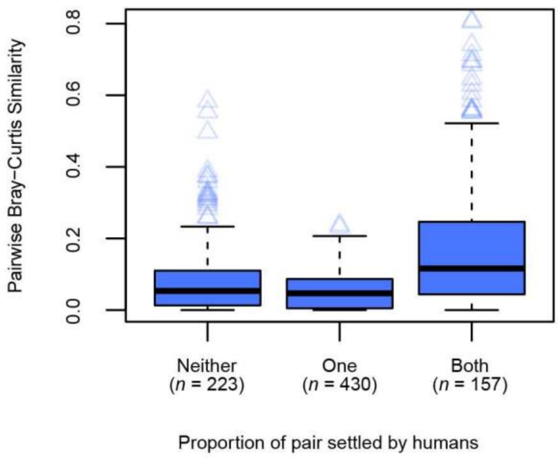

Figure S13. Pairwise Bray-Curtis comparisons for the standardization-2 dataset grouped according to whether neither, one, or both islands were settled during a time interval. *n* = number of individual pairwise comparisons. The black horizonal lines represent the medians and the box areas represent the first and third quantiles. The whiskers of the boxplot extend to the last data points within 1.5 times the range from first to third quantile (interquartile range of the box). The median similarity of individual pairwise comparisons where neither, one, or both locations were settled by humans was 0.05, 0.05, and 0.12 respectively.

Table S5. Most abundant taxa before and after human settlement.

**Before settlement standardization-1**		**Before settlement standardization-2**		**After settlement standardization-1**		**After settlement standardization-2**	
**Taxon name**	**mean %**	**Taxon name**	**mean %**	**Taxon name**	**mean %**	**Taxon name**	**mean %**
Euphorbiaceae	6	Euphorbiaceae	8	Cyperaceae	16	Cyperaceae	19
Poaceae	6	*Pandanus*	7	Poaceae	10	Poaceae	10
Acrostichum	5	Cyperaceae	7	*Pandanus*	5	*Pandanus*	8
Pandanus tectorius	4	Poaceae	6	Euphorbiaceae	5	Euphorbiaceae	8
Pandanus	4	*Acrostichum*	5	*Excoecaria*	4	*Acrostichum*	4
Myrtaceae	4	Myrtaceae	4	*Acrostichum*	4	*Pteridium esculentum*	3
Macaranga	3	*Pandanus tectorius*	4	*Pteridium esculentum*	3	Aspleniaceae	3
Cyperaceae	3	*Macaranga*	3	*Blechnum*	3	*Macaranga*	2
Metrosideros	3	*Metrosideros*	3	*Pandanus tectorius*	3	Casuarinaceae	2
Scirpus	3	*Trema*	3	*Macaranga*	2	Arecaceae	2

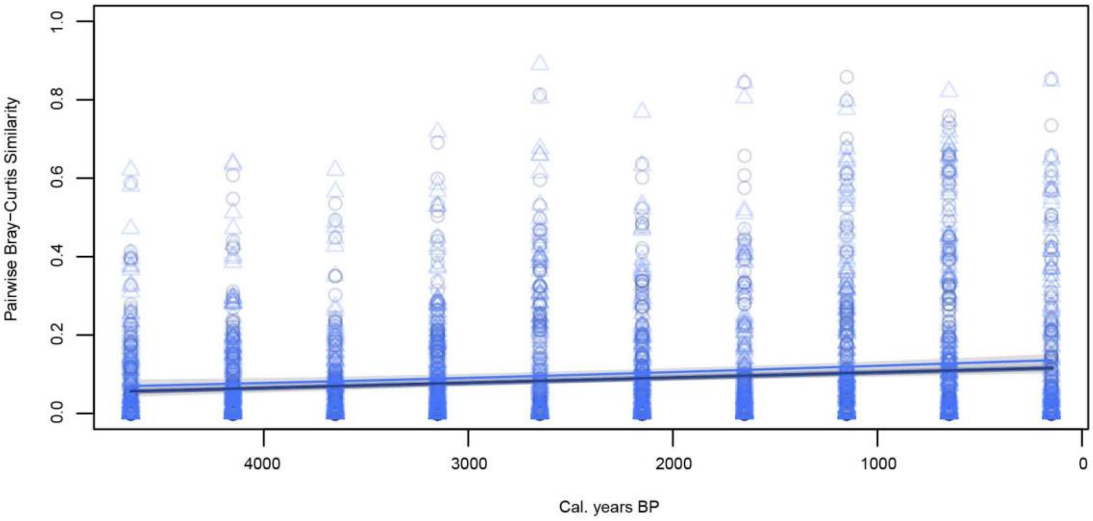

Figure S14. Non-parametric regressions were fitted using smoothing splines deploying package *npreg* (Helwig, 2020) with function ss (fit a smoothing spline). Similarity trends between taxa within the same rank. Smoothing splines displaying pairwise Bray-Curtis similarity scores where only taxa within the same rank are compared among all 15 sites on 13 islands, over the past 5000 cal. years. 1= indicates greatest similarity and 0= lowest similarity. The dark blue line represents standardization-1 and the lighter blue line represents standardization-2. Open circles represent standardization-1 datapoints and open triangles represent standardization-2 datapoints. The gray shaded area represents a confidence interval of 95%. The standardization-1 dataset includes 1887 individual pairwise comparisons (family=762, genus=810, species=315). The standardization-2 dataset includes 1874 individual pairwise comparisons (family= 810, genus=810, species=254).

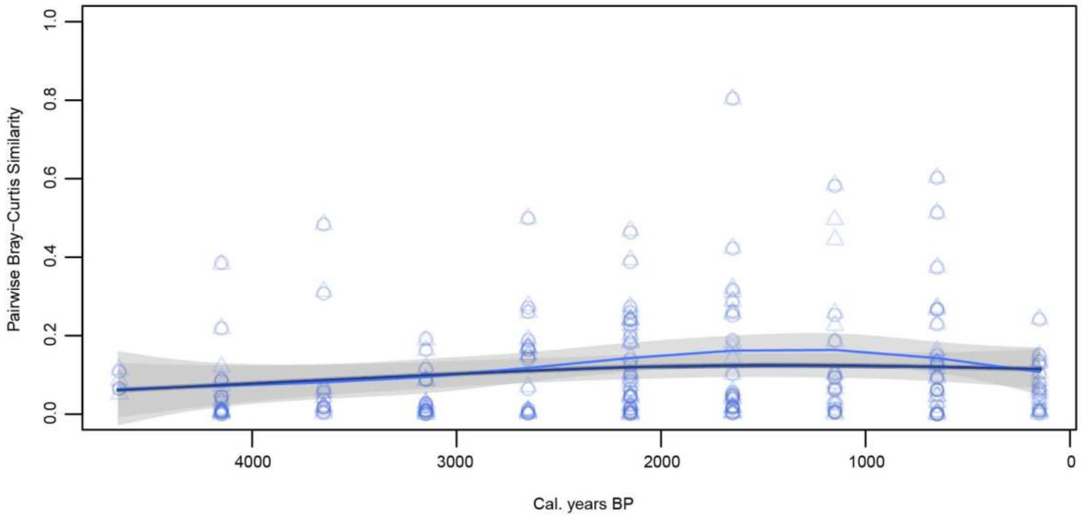

Figure S15. Non-parametric regressions were fitted using smoothing splines deploying package *npreg* (Helwig, 2020) with function ss (fit a smoothing spline). Similarity trends excluding pollen assemblages with <300 taxa. Smoothing splines displaying pairwise Bray-

Curtis similarity scores for taxa within pollen samples with counts >300, among all 15 sites on 13 islands, over the past 5000 cal. years. 1= indicates greatest similarity and 0= lowest similarity. The dark blue line represents standardization-1 and the lighter blue line represents standardization-2. Open circles represent standardization-1 datapoints and open triangles represent standardization-2 datapoints. The gray shaded area represents a confidence interval of 95%. Both datasets contain 158 individual pairwise comparison values.

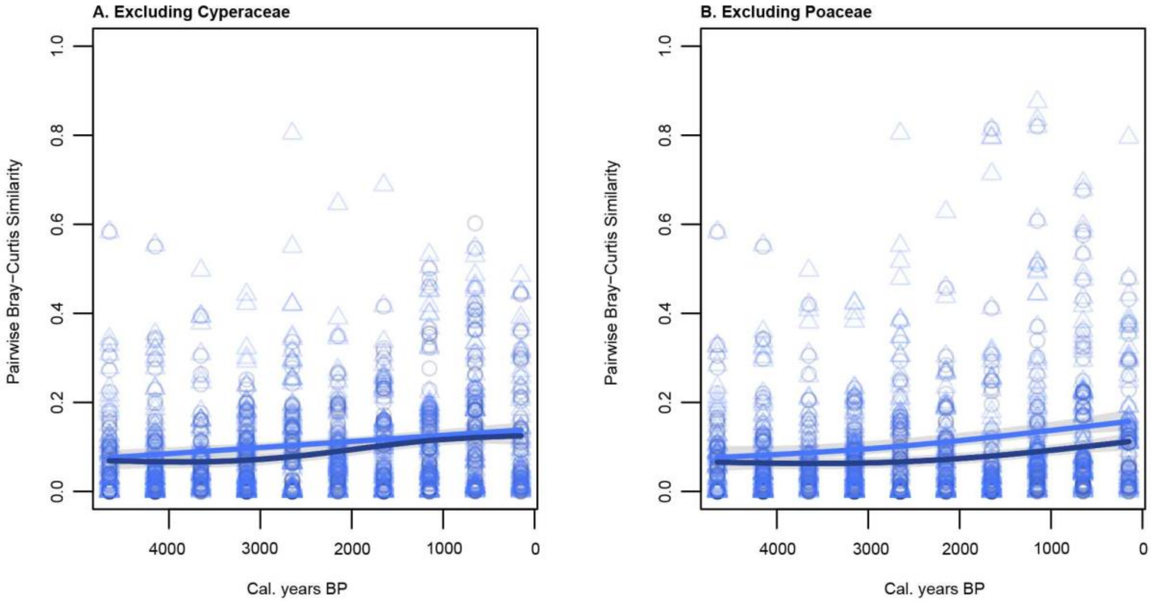

Figure S16. Similarity trends excluding Cyperaceae and Poaceae. Non-parametric regressions were fitted using smoothing splines deploying package *npreg* (Helwig, 2020) with function ss (fit a smoothing spline). Smoothing splines displaying pairwise Bray-Curtis similarity scores excluding A. Cyperaceae, and B. Poaceae, among all 15 sites on 13 islands, over the past 5000 cal. years. 1= indicates greatest similarity and 0= lowest similarity. The dark blue line represents standardization-1 and the lighter blue line represents standardization-2. Open circles represent standardization-1 datapoints and open triangles represent standardization-2 datapoints. The gray shaded area represents a confidence interval of 95%.

References

ANDERSON, A. & CLARK, G. 1999. The age of Lapita settlement in Fiji. *Archaeology in Oceania,* 34**,** 31-39.

BLAAUW, M. & CHRISTEN, J. A. 2011. Flexible Paleoclimate Age-Depth Models Using an Autoregressive Gamma Process. *Bayesian Analysis,* 6**,** 457-474.

BURLEY, D., EDINBOROUGH, K., WEISLER, M. & ZHAO, J. X. 2015. Bayesian Modeling and Chronological Precision for Polynesian Settlement of Tonga. *Plos One,* 10.

FLENLEY, J. R., HANNAN, C. T. & FARELLY, M. J. 1999. Final report on the stratigraphy and palynology of swamps on the islands of Ha'afeva and Foa, Ha'apai, Tonga. *Geography Programme Miscellaneous Publication.* Palmerston, New Zealand: Massey University.

GOOGLE EARTH PRO. 2023. *Google Earth Pro V 7.3.6.9345* [Online]. [Accessed 6th February 2023].

HAYWARD, B. 1976. Geology of the Whitianga Group, Great Mercury Island - Part I. Coroglen Subgroup stratigraphy. *Tane,* 22**,** 5-14.

HELWIG, N. 2020. Multiple and generalized nonparametric regression. *P. Atkinson, S. Delamont, A. Cernat, JW Sakshaug, & RA Williams (Eds.).* SAGE Research Methods Foundations.

HOGG, A. G., HEATON, T. J., HUA, Q., PALMER, J. G., TURNEY, C. S. M., SOUTHON, J., BAYLISS, A., BLACKWELL, P. G., BOSWIJK, G., BRONK RAMSEY, C., PEARSON, C., PETCHEY, F., REIMER, P., REIMER, R. & WACKER, L. 2020. SHCal20 Southern Hemisphere Calibration, 0–55,000 Years cal BP. *Radiocarbon,* 62**,** 759-778.

HOLDAWAY, S. J., EMMITT, J., FUREY, L., JORGENSEN, A., O'REGAN, G., PHILLIPPS, R., PREBBLE, M., WALLACE, R. & LADEFOGED, T. N. 2019. Māori settlement of New Zealand: The Anthropocene as a process. *Archaeology in Oceania,* 54**,** 17-34.

HOPE, G. & SPRIGGS, M. 1982. A preliminary pollen sequence from Aneityum Island, Southern Vanuatu. *Bulletin of the Indo-Pacific Prehistory Association*.

HOPE, G., STEVENSON, J. & SOUTHERN, W. 2009. Vegetation histories from the Fijian Islands: Alternative records of human impact. *Early Prehistory of Fiji,* 31**,** 63-86.

HUNT, T. L. & LIPO, C. P. 2006. Late colonization of Easter Island. *Science,* 311**,** 1603-1606.

KENNETT, D., ANDERSON, A., PREBBLE, M., CONTE, E. & SOUTHON, J. 2006. Prehistoric Human Impacts on Rapa, French Polynesia. *Antiquity,* 80.

LEACH, H. M. & GREEN, R. C. 1989. New information for the Ferry Berth site, Mulifanua, western Samoa. *The Journal of the Polynesian Society,* 98**,** 319-329.

NUNN, P. D., KUMAR, L., ELIOT, I. & MCLEAN, R. F. 2016. Classifying Pacific islands. *Geoscience Letters,* 3.

PETCHEY, F., SPRIGGS, M., BEDFORD, S., VALENTIN, F. & BUCKLEY, H. 2014. Radiocarbon dating of burials from the Teouma Lapita cemetery, Efate, Vanuatu. *Journal of Archaeological Science,* 50**,** 227-242.

PREBBLE, M., ANDERSON, A. J., AUGUSTINUS, P., EMMITT, J., FALLON, S. J., FUREY, L. L., HOLDAWAY, S. J., JORGENSEN, A., LADEFOGED, T. N., MATTHEWS, P. J., MEYER, J.-Y., PHILLIPPS, R., WALLACE, R. & PORCH, N. 2019. Early tropical crop production in marginal subtropical and temperate Polynesia. *Proceedings of the National Academy of Sciences***,** 201821732.

SAND, C. 1997. The chronology of Lapita ware in New Caledonia. *Antiquity,* 71**,** 539-547.

SOUTHERN, W. 1986. *The Late Quaternary environmental history of Fiji.* Unpublished PhD thesis, Australian National University.

STEVENSON, J. 2004. A late-Holocene record of human impact from the southwest coast of New Caledonia. *Holocene,* 14**,** 888-898.

STEVENSON, J. & DODSON, J. R. 1995. Palaeoenvironmental evidence for human settlement of New Caledonia. *Archaeology in Oceania,* 30**,** 36-41.

STEVENSON, J., DODSON, J. R. & PROSSER, I. P. 2001. A late Quaternary record of environmental change and human impact from New Caledonia. *Palaeogeography Palaeoclimatology Palaeoecology,* 168**,** 97-123.

STRANDBERG, N. A., EDWARDS, M., ELLISON, J. C., STEINBAUER, M. J., WALENTOWITZ, A., FALL, P. L., SEAR, D., LANGDON, P., CRONIN, S., CASTILLA-BELTRÁN, A.,

CROUDACE, I. W., PREBBLE, M., GOSLING, W. D. & NOGUÉ, S. 2023. Influences of sea level changes and volcanic eruptions on Holocene vegetation in Tonga. *Biotropica,* 55**,** 816-827.

WEIGELT, P., JETZ, W. & KREFT, H. 2013. Bioclimatic and physical characterization of the world's islands. *Proceedings of the National Academy of Sciences*, 110**,** 15307-15312.

WILMSHURST, J. M., HUNT, T. L., LIPO, C. P. & ANDERSON, A. J. 2011. High-precision radiocarbon dating shows recent and rapid initial human colonization of East Polynesia. *Proceedings of the National Academy of Sciences of the United States of America,* 108**,** 1815-1820.
