# Supplementary material for: Floristic homogenization of South Pacific islands commenced with human arrival
Source: Nat Ecol Evol. 2024 Jan 15;8(3):511–8. doi: 10.1038/s41559-023-02306-3 (PMC10927543; doi:10.1038/s41559-023-02306-3)
Supplement: Supplementary file 2 — Reporting Summary [file 41559_2023_2306_MOESM2_ESM.pdf]

nature portfolio

Corresponding author(s): Nichola Strandberg, Manuel Steinbauer, and Sandra Nogué

Last updated by author(s): Nov 30, 2023

Reporting Summary

Nature Portfolio wishes to improve the reproducibility of the work that we publish. This form provides structure for consistency and transparency in reporting. For further information on Nature Portfolio policies, see our Editorial Policies and the Editorial Policy Checklist.

Statistics

For all statistical analyses, confirm that the following items are present in the figure legend, table legend, main text, or Methods section.

n/a	Confirmed	
☐	☒	The exact sample size (*n*) for each experimental group/condition, given as a discrete number and unit of measurement
☐	☒	A statement on whether measurements were taken from distinct samples or whether the same sample was measured repeatedly
☐	☒	The statistical test(s) used AND whether they are one- or two-sided<br>*Only common tests should be described solely by name; describe more complex techniques in the Methods section.*
☐	☒	A description of all covariates tested
☒	☐	A description of any assumptions or corrections, such as tests of normality and adjustment for multiple comparisons
☐	☒	A full description of the statistical parameters including central tendency (e.g. means) or other basic estimates (e.g. regression coefficient) AND variation (e.g. standard deviation) or associated estimates of uncertainty (e.g. confidence intervals)
☒	☐	For null hypothesis testing, the test statistic (e.g. *F*, *t*, *r*) with confidence intervals, effect sizes, degrees of freedom and *P* value noted<br>*Give P values as exact values whenever suitable.*
☒	☐	For Bayesian analysis, information on the choice of priors and Markov chain Monte Carlo settings
☒	☐	For hierarchical and complex designs, identification of the appropriate level for tests and full reporting of outcomes
☒	☐	Estimates of effect sizes (e.g. Cohen's *d*, Pearson's *r*), indicating how they were calculated

Our web collection on statistics for biologists contains articles on many of the points above.

Software and code

Policy information about availability of computer code

Data collection	RStudio 2023.03.1 and R 4.2.1. were used.
Data analysis	RStudio 2023.03.1 and R 4.2.1. were used.

For manuscripts utilizing custom algorithms or software that are central to the research but not yet described in published literature, software must be made available to editors and reviewers. We strongly encourage code deposition in a community repository (e.g. GitHub). See the Nature Portfolio guidelines for submitting code & software for further information.

Data

Policy information about availability of data

All manuscripts must include a data availability statement. This statement should provide the following information, where applicable:

- Accession codes, unique identifiers, or web links for publicly available datasets
- A description of any restrictions on data availability
- For clinical datasets or third party data, please ensure that the statement adheres to our policy

Data and R scripts are available at https://doi.org/10.6084/m9.figshare.22794191 and https://github.com/nastrandberg/Biotic_homogenisation.git

Research involving human participants, their data, or biological material

Policy information about studies with human participants or human data. See also policy information about sex, gender (identity/presentation), and sexual orientation and race, ethnicity and racism.

Reporting on sex and gender	N/A
Reporting on race, ethnicity, or other socially relevant groupings	N/A
Population characteristics	N/A
Recruitment	N/A
Ethics oversight	N/A

Note that full information on the approval of the study protocol must also be provided in the manuscript.

Field-specific reporting

Please select the one below that is the best fit for your research. If you are not sure, read the appropriate sections before making your selection.

- [ ] Life sciences
- [ ] Behavioural & social sciences
- [x] Ecological, evolutionary & environmental sciences

For a reference copy of the document with all sections, see nature.com/documents/nr-reporting-summary-flat.pdf

Ecological, evolutionary & environmental sciences study design

All studies must disclose on these points even when the disclosure is negative.

Study description	We tested the similarity of fossil pollen data through time and between different locations. Prior to analysis, pollen data with uncertain identifications were removed. This process is shown in the R code and described in the methods.
Research sample	Some datasets were accessed via the Neotoma database https://www.neotomadb.org/ wheras others were accessed via publications and digitization. The pollen data was from islands in the South Pacific and we aimed to use the longest pollen records.
Sampling strategy	We included all pollen records >3300 years long and with higher pollen counts than 60 per sample as we deemed this sample size to be rather small and not representative.
Data collection	Data was collected from published papers and from online repositories by Nichola Strandberg.
Timing and spatial scale	The spatial scale of the study is from -13.9093 to -36.61015 latitude and 166.55 to -109.435 longitude in the South Pacific Ocean between New Caledonia and Rapa Nui (Easter Island). The oldest pollen sample is from 4991 calibrated years BP to the most recent from 2018 CE. Nichola Strandberg accessed these datasets between 2020 and 2023.
Data exclusions	A dataset with pollen counts <60 was excluded since we considered this to be low compared to other datasets. Data points older than 5000 years were excluded since there was a lack of data points therefore decreasing the opportunity for similarity comparisons. This is explained in the manuscript methods section.
Reproducibility	The R code is well organized and has comments at each stage to explain what was done. The methods section of the manuscript also summarizes this.
Randomization	This is not relevant since no data was required to be randomized since we only used pollen data.
Blinding	Blinding was not required in the study since we just used pollen data.

Did the study involve field work? [ ] Yes [x] No

Reporting for specific materials, systems and methods

We require information from authors about some types of materials, experimental systems and methods used in many studies. Here, indicate whether each material, system or method listed is relevant to your study. If you are not sure if a list item applies to your research, read the appropriate section before selecting a response.

Materials & experimental systems

n/a	Involved in the study
☒	☐ Antibodies
☒	☐ Eukaryotic cell lines
☒	☐ Palaeontology and archaeology
☒	☐ Animals and other organisms
☒	☐ Clinical data
☒	☐ Dual use research of concern
☒	☐ Plants

Methods

n/a	Involved in the study
☒	☐ ChIP-seq
☒	☐ Flow cytometry
☒	☐ MRI-based neuroimaging
